# Effectiveness and safety of denosumab monotherapy in patients with primary hyperparathyroidism: A retrospective study

**DOI:** 10.1097/MD.0000000000044882

**Published:** 2025-10-03

**Authors:** Akira Okada, Ryuta Baba, Yuria Ishibashi, Yu Otagaki, Takaya Kodama, Gentaro Egusa, Gaku Nagano, Tsuguka Matsuda, Noboru Hattori, Haruya Ohno

**Affiliations:** a Department of Molecular and Internal Medicine, Graduate School of Biomedical and Health Science, Hiroshima University, Hiroshima, Japan.

**Keywords:** denosumab, hypercalcemia, osteoporosis, primary hyperparathyroidism, retrospective study

## Abstract

The appropriate treatment of osteoporosis and hypercalcemia using medications is important in patients with primary hyperparathyroidism (PHPT) who do not undergo parathyroidectomy. Denosumab effectively treats osteoporosis in patients with PHPT and improves hypercalcemia in the short term after administration. However, the medium-term effects of denosumab monotherapy on serum calcium levels and its safety in patients with PHPT remain unclear. In this study, we aimed to determine the medium-term effect of denosumab on serum calcium levels and bone mineral density (BMD) in patients. In this retrospective study, we compared serum calcium levels in patients with PHPT who did not undergo parathyroidectomy before denosumab monotherapy, 3 and 6 months after administration, respectively. Furthermore, we analyzed whether BMD increased 1 year after administration. Serum calcium levels in 15 patients with PHPT did not significantly change before (median serum calcium level, 10.8 mg/dL) or 6 months after denosumab administration (10.8 mg/dL). However, among 5 patients who visited the hospital for treatment of different diseases and whose serum calcium levels were measured 3 months after administration, their serum calcium levels significantly decreased after 3 months (from 10.6 to 9.8 mg/dL). No patient developed hypocalcemia. The BMD in the lumbar spine increased 12 months after denosumab administration (from 0.707 to 0.784 g/cm^2^). These results suggest that denosumab administration alone is safe for hypocalcemia and effective for treating osteoporosis in patients with PHPT. In addition, it can be a good treatment option for patients who do not undergo parathyroidectomy.

## 1. Introduction

Primary hyperparathyroidism (PHPT) is characterized by excessive secretion of parathyroid hormone. Furthermore, its oversecretion causes hypercalcemia, hypophosphatemia, renal impairment, and osteoporosis. PHPT is a common disease with a prevalence rate of 1 patient per 2500–5000 people. Its origin is a benign adenoma in 80% of patients, whereas others include hyperplasia in multiple endocrine adenomatoses and malignant tumors.^[1]^

Parathyroidectomy is the standard treatment for PHPT; however, drug therapy is necessary to manage complications if it is not appropriate or if the patient does not consent to surgery.^[2]^ Changes in bone mineral density (BMD) in patients with PHPT with or without parathyroidectomy reveal that BMD increases immediately after parathyroidectomy and in the femoral neck and distal radius in patients without parathyroidectomy, it starts to significantly decrease 10 years after the beginning of the observation.^[3]^ Therefore, treatment of osteoporosis is important when drug therapy is selected for PHPT.

Denosumab is a monoclonal antibody against receptor activator of nuclear factor kappa-B ligand (RANKL) that significantly increases BMD in postmenopausal women with osteoporosis and PHPT.^[4,5]^ In these studies, patients were prescribed calcium or vitamin D along with denosumab. Denosumab is recommended to be prescribed with adequate calcium and vitamin D supplementation when used for general osteoporosis as it may induce hypocalcemia via strong inhibition of osteoclastic bone resorption.^[6]^ Denosumab is therapeutically effective against severe hypercalcemia in metastatic bone tumors.^[7]^ In addition, 60 mg of denosumab improves hypercalcemia within 7 days in patients with PHPT whose serum calcium levels are >14 mg/dL or who have symptomatic hypercalcemia.^[8]^

Furthermore, denosumab is effective in the short-term management of severe hypercalcemia in addition to osteoporosis. However, the effect of denosumab monotherapy on serum calcium levels in patients with PHPT for medium-term management is unclear. In addition, to the best of our knowledge, no study has been conducted on the effectiveness of denosumab monotherapy on bone turnover in patients with PHPT, whereas some studies reported its effects in postmenopausal women and patients treated with glucocorticoids.^[9,10]^ Elucidating the effects of denosumab alone in patients with PHPT regarding serum calcium and BMD may help us understand the benefits and risks of hypocalcemia with denosumab alone, leading to its appropriate use for patients with PHPT. Therefore, we aimed to determine the medium-term effect of denosumab on serum calcium levels and BMD in patients.

## 2. Methods

### 2.1. Study design, patient selection, and data source

We included patients with PHPT who did not undergo parathyroidectomy and were started on denosumab treatment for osteoporosis at the Department of Endocrinology and Diabetic Medicine at Hiroshima University Hospital between 2013 and 2022. We retrospectively and statistically compared the albumin-corrected serum calcium levels before the first and second denosumab administration 6 months later. In addition, we investigated the changes in serum phosphorus levels, intact parathyroid hormone (iPTH) levels, alkaline phosphatase (ALP) levels, and estimated glomerular filtration rate (eGFR) at 6 months. The change in serum calcium levels from baseline of 3 months after administration among patients whose calcium levels were measured at the time were evaluated. We also assessed the differences in baseline calcium levels, ALP levels, and eGFR between patients who underwent a blood test, 3 months after administration, and those who did not. BMDs in the lumbar spine, distal radius, and femoral neck were measured 1 year after administration and compared with those before administration.

We excluded patients taking medications that could change serum calcium levels, including vitamin D preparations during observation, patients who underwent parathyroidectomy before 6 months of follow-up, and patients whose calcium levels were not measured before or 6 months after denosumab administration. All data were collected from the electronic medical records.

### 2.2. Clinical data

The patients’ clinical data included age; sex; serum calcium (mg/dL), albumin (g/dL), phosphorus (mg/dL), iPTH (pg/mL), and ALP (U/L) levels; eGFR (mL/min/1.73 m^2^); and BMD (g/cm^2^). Serum calcium, albumin, phosphorus, iPTH, ALP levels, and eGFR were measured using standard commercial assays. When patients had hypoalbuminemia (serum albumin level <4.0 mg/dL), we calculated the corrected calcium level for serum calcium levels. The corrected calcium level was calculated as follows: corrected calcium = total serum calcium concentration + (4.0—serum albumin). The ALP measurement method at Hiroshima University Hospital was used by the Japanese Society of Clinical Chemistry (JSCC) before 2021. ALP has been measured by both JSCC and International Federation of Clinical Chemistry and Laboratory Medicine since 2021. Therefore, we used the ALP value in JSCC, which was involved a larger number of patients. BMD was measured using dual-energy X-ray absorptiometry using Hologic QDR-1000 (Hologic Inc., Marlborough, MA). The lumbar spine BMD was measured at the lumbar vertebrae 2 to 4. Distal radius BMD was measured at the distal one-third of the radius. The distal radius and femoral neck BMDs were defined as the right and left mean values, respectively.

### 2.3. Statistical analyses

Continuous variables are presented as medians with interquartile ranges (IQR). Categorical variables are presented as frequencies (n) and percentages (%). When the patients’ inspection items were not measured at the targeted time, their data were excluded from the analysis. The Wilcoxon signed-rank test was used to determine whether the variables significantly differed between baseline and after administration. We used an independent *t* test to compare baseline variables between the 2 groups of patients. Statistical significance was set at *P* < .05 for both tests. Statistical analyses were performed using IBM SPSS Statistics for Windows (version 27.0; SPSS Inc. Corp., Armonk, NY).

### 2.4. Ethics approval

This study was approved by the Ethical Committee for Clinical Research of Hiroshima University (Hiroshima, Hiroshima, Japan) and conducted according to the principles of the Declaration of Helsinki. Informed consent was waived due to the retrospective nature of the study.

### 2.5. Data availability statement

The datasets analyzed during the current study are available from the corresponding author on reasonable request.

## 3. Results

### 3.1. Baseline characteristics of the patients

Of the 29 patients with PHPT enrolled in this retrospective study, we excluded 7 patients who were taking medicines that caused serum calcium changes (6 patients were taking precipitated calcium carbonate with cholecalciferol and magnesium, and 1 patient was taking cinacalcet hydrochloride), 3 patients who underwent parathyroidectomy within 6 months after the first denosumab administration, and 4 patients whose serum calcium levels were not measured before and 6 months after denosumab administration. Subsequently, 15 patients with PHPT (11 women and 4 men) met the inclusion criteria. Table 1 shows the baseline demographic and clinical characteristics of the patients. The median patient age was 73 (IQR, 61–76) years. The median calcium level was 10.8 (IQR, 10.3–11.0) mg/dL, phosphorus level 2.6 (IQR, 2.3–3.1) mg/dL, iPTH level 120 (IQR, 92–166) pg/mL, ALP level 316 (IQR, 230–366) U/L, and eGFR 68 (IQR, 61–81) mL/min/1.73 m^2^. The median BMDs were 0.707 (IQR, 0.628–0.810) g/cm^2^ in the lumbar spine, 0.448 (IQR, 0.376–0.581) g/cm^2^ in the distal radius, and 0.447 (IQR, 0.404–0.603) g/cm^2^ in the femoral neck.

**Table 1:** Baseline characteristics of the patients.

Background	n = 15
Age, years, median (IQR)	73 (61–76)
Female, n (%)	11 (73.3)
Serum calcium, mg/dL, median (IQR)	10.8 (10.3–11.0)
Serum phosphorus, mg/dL, median (IQR)	2.6 (2.3–3.1)
iPTH, pg/mL, median (IQR)	120 (92–166)
ALP (JSCC), U/L, median (IQR)	316 (230–366)
eGFR, mL/min/1.73 m^2^, median (IQR)	68 (61–81)
Lumbar BMD, g/cm^2^, median (IQR)	0.707 (0.628–0.810)
Distal radius BMD, g/cm^2^, median (IQR)	0.448 (0.376–0.581)
Femur BMD, g/cm^2^, median (IQR)	0.447 (0.404–0.603)

ALP *=* alkaline phosphatase, BMD *=* bone mineral density, eGFR *=* estimated glomerular filtration rate, iPTH *=* intact parathyroid hormone, IQR *=* interquartile range, JSCC *=* Japanese Society of Clinical Chemistry.

### 3.2. Changes in clinical values after denosumab administration

Table 2 shows the patients’ calcium, phosphorus, iPTH and ALP levels, and eGFR at baseline and 6 months after denosumab administration. There was no significant difference in calcium levels between baseline and after denosumab administration (baseline: median, 10.8 [IQR, 10.3–11.0] mg/dL; after denosumab administration: median, 10.8 [IQR, 10.7–11.0] mg/dL; *P* = .161). The median change in serum calcium levels (Hodges–Lehmann estimate) was 0.15 mg/dL (95% CI: −0.10 to 0.40). There were no significant changes in the phosphorus levels (*P* = .408), iPTH levels (*P* = .155), and eGFR (*P* = .401). ALP levels significantly decreased after administration (baseline: median, 316 [IQR, 230–366] U/L; after denosumab administration: median, 214 [IQR, 166–313] U/L; *P* = .038). The median change in ALP levels (Hodges–Lehmann estimate) was −75 U/L (95% CI: −362 to −3).

**Table 2:** Variable comparisons between baseline and 6 months after denosumab administration.

	Before denosumab administration	6 months after denosumab administration	*P* value
Serum calcium, mg/dL, median (IQR) (n = 15)	10.8 (10.4–11.0)	10.8 (10.7–11.0)	.161
Serum phosphorus, mg/dL, median (IQR) (n = 13)	2.6 (2.3–3.1)	2.6 (2.1–3.0)	.408
iPTH, pg/mL, median (IQR) (n = 11)	120 (92–166)	149 (88–203)	.155
ALP (JSCC), U/L, median (IQR) (n = 10)	316 (230–366)	214 (166–313)	.038
eGFR, mL/min/1.73 m^2^, median (IQR) (n = 13)	68 (61–81)	72 (60–76)	.401

The *P*-values were determined using the Wilcoxon signed-rank test.

ALP *=* alkaline phosphatase, eGFR *=* estimated glomerular filtration rate, iPTH *=* intact parathyroid hormone, IQR *=* interquartile range, JSCC *=* Japanese Society of Clinical Chemistry.

Table 3 shows the median BMDs of the lumbar spine, distal radius, and femoral neck at baseline and 12 months after denosumab administration. Lumbar spine BMD significantly increased after denosumab administration (baseline: median, 0.707 [IQR, 0.628–0.810] g/cm^2^; denosumab administration: median, 0.784 [IQR, 0.627–0.886] g/cm^2^; *P* = .036). The median improvement in lumber spine BMD (Hodges–Lehmann estimate) was 0.045 g/cm^2^ (95% CI: 0.010–0.077). Neither the distal radius nor the femoral neck BMD increased considerably (distal radius baseline: 0.448 [IQR, 0.376–0.581] g/cm^2^; after denosumab administration: 0.452 [IQR, 0.380–0.600] g/cm^2^; *P* = .31 and femoral neck baseline: 0.447 [IQR, 0.404–0.603] g/cm^2^; after denosumab administration: 0.462 [IQR, 0.429–0.623] g/cm^2^; *P* = .063). The median change in BMD (Hodges–Lehmann estimate) was 0.004 g/cm^2^ at the distal radius (95% CI: −0.009 to 0.019), and was 0.015 g/cm^2^ at the femoral neck (95% CI: −0.003 to 0.025).

**Table 3:** BMD comparisons between baseline and 12 months after denosumab administration.

	Before denosumab administration	12 months after denosumab administration	*P* value
Lumbar BMD, g/cm^2^, median (IQR) (n = 8)	0.707 (0.628–0.810)	0.784 (0.627–0.886)	.036
Distal radius BMD, g/cm^2^, median (IQR) (n = 7)	0.448 (0.376–0.581)	0.452 (0.380–0.600)	.31
Femur BMD, g/cm^2^, median (IQR) (n = 7)	0.447 (0.404–0.603)	0.462 (0.429–0.623)	.063

The *P*-values were determined using the Wilcoxon signed-rank test.

BMD = bone mineral density, IQR *=* interquartile range.

### 3.3. Changes in serum calcium levels 3 months after denosumab administration

**Figure 1.**
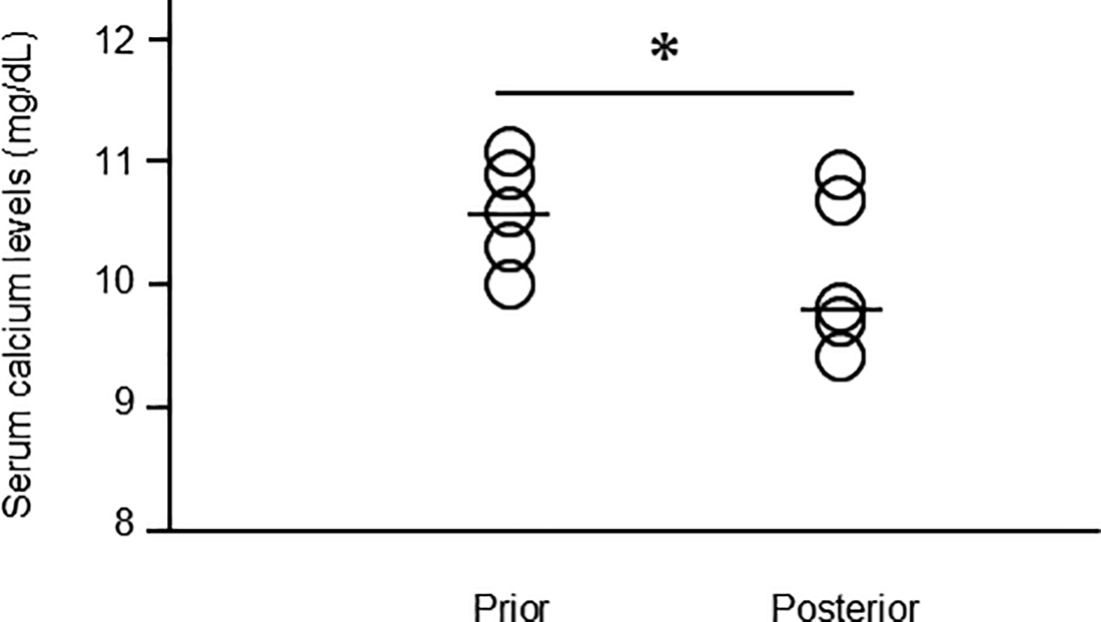
Changes in serum calcium levels 3 months after denosumab administration. Changes in serum calcium levels before and 3 months after denosumab administration. The median serum calcium level before denosumab was 10.8 mg/dL. Three months after denosumab administration, it significantly decreased to 9.8 mg/dL. The *P*-value was determined using the Wilcoxon signed-rank test. ^+^Differences at *P* < .05 is considered statistically significant. *Prior* before denosumab administration, *Posterior* 3 months after denosumab administration.

Figure 1 shows the changes in serum calcium levels in 5 patients whose calcium levels were measured 3 months after denosumab administration. The median calcium level at baseline was 10.6 (IQR, 10.3–10.9) mg/dL, whereas it decreased to 9.8 (IQR, 9.7–10.7) mg/dL 3 months after denosumab administration (*P* = .042). None of the patients presented with hypocalcemia during the study period. Calcium levels in all 5 patients were measured 3 months after administration as they visited Hiroshima University Hospital and underwent blood tests for diseases other than PHPT (Table S1, Supplemental Digital Content, https://links.lww.com/MD/Q184). In all the patients, the Department of Endocrinology and Diabetic Medicine followed up their calcium levels 6 months later for the first time after denosumab administration. There were no significant differences in baseline serum calcium and ALP levels and eGFR between these 5 patients and the other 10 patients (Table S2, Supplemental Digital Content, https://links.lww.com/MD/Q184).

## 4. Discussion

In this study, we aimed to elucidate the effects of denosumab on serum calcium levels and BMD in patients with PHPT. The results showed that the risk of hypocalcemia was low and the lumbar spine BMD increased when denosumab was administered alone to patients with mild PHPT who did not undergo surgery.

Denosumab binds RANKL and inhibits the interaction between RANKL and RANK, leading to the suppression of bone absorption.^[11]^ The decline in bone absorption transiently decreases the serum calcium level.^[6,12]^ Therefore, denosumab can improve hypercalcemia in patients with metastatic bone tumors or PHPT.^[7,8]^ In this study, the administration of denosumab alone to patients with PHPT decreased serum calcium levels after 3 months. They returned to almost the same levels as the baseline after 6 months, when the patients were administered denosumab for the second time. Miyaoka et al revealed that serum calcium levels decreased most significantly 7 days after denosumab administration. Furthermore, there was no significant difference in serum calcium levels between the baseline and 6 months after administration when patients with primary osteoporosis were treated with concomitant use of denosumab and precipitated calcium carbonate with cholecalciferol and magnesium.^[13]^ This finding was consistent with our results, which showed no significant change between baseline and 6 months after denosumab administration. In patients with PHPT, denosumab and 50 mcg of vitamin D daily decreased serum calcium levels after administration, but the serum calcium levels returned to almost the same levels as the baseline after 4 months.^[14]^ These results do not differ vastly from our results regarding the decrease in serum calcium levels. These results suggest that the serum calcium decrease does not accumulate or intensify if denosumab is used semiannually. Serum calcium levels decreased after 3 months in patients whose calcium levels were measured at that time. How denosumab influenced serum calcium levels in the other patients is unclear. High ALP level and low eGFR were indicated as predictors of hypocalcemia after denosumab administration.^[15,16]^ In our study, neither baseline ALP level nor eGFR significantly differed between patients whose serum calcium levels were measured 3 months after denosumab administration and those whose serum calcium levels were not measured at that time. Therefore, it is possible that serum calcium levels decreased 3 months after denosumab administration in the latter patients. However, none of the patients were diagnosed with hypocalcemia. Consequently, we believe that patients with PHPT who receive treatment with denosumab alone are at a low risk of developing hypocalcemia.

Subclinical vertebral fractures are common in asymptomatic PHPT.^[17]^ ALP is involved in bone formation and accelerates bone metabolism conditions, such as PHPT.^[18,19]^ Denosumab treatment decreases ALP levels and improves bone density in postmenopausal women with PHPT.^[5]^ Similarly, denosumab significantly decreased ALP levels and improved the BMD of the lumbar spine in our study (Table 3). The BMD in the distal radius and femoral neck showed no significant changes; however, the median BMD slightly increased. Therefore, our study indicates that denosumab suppresses bone metabolism turnover and improves BMD, similar to previous reports in patients with PHPT.^[4,5]^ In such previous studies on osteoporosis treatment, denosumab was prescribed with calcium or vitamin D. To the best of our knowledge, this study is the first in which only denosumab was used for osteoporosis treatment in patients with PHPT. Bisphosphonates, estrogen therapy, and denosumab increased BMD in patients.^[20]^ Therefore, denosumab monotherapy is a convenient treatment option for patients with PHPT who cannot solely manage their internal medications.

This study has some limitations. First, considering this study included only patients with PHPT who had not undergone surgery, serum calcium levels did not exceed the upper limit of normal by 1 mg/dL in a large number of patients. However, patients with higher serum calcium levels have a lower risk of developing hypocalcemia after denosumab administration. Second, only 3 of 15 patients’ eGFR were <60 mL/min/1.73 m^2^, indicating chronic kidney disease (CKD). Denosumab tends to decrease serum calcium levels more in patients with CKD because of renal calcium reabsorption disorder, vitamin D activation disorder in the kidney, and intestinal calcium absorption disorder.^[21,22]^ Therefore, the risk of hypocalcemia might be higher in patients with CKD than in patients in this study. Moreover, we cannot exclude the possibility that renal function may have influenced serum calcium levels despite including few patients with CKD. However, no significant decrease in serum calcium was observed at 6 months, and no cases of hypocalcemia were reported among patients with CKD during the observation period. On the other hand, denosumab is effective in improving BMD in patients with CKD or undergoing dialysis.^[23–25]^ We believe that denosumab should be considered as a treatment option for patients with PHPT and CKD who cannot undergo parathyroidectomy. However, careful monitoring of serum calcium levels is warranted during treatment given the potential risk of hypocalcemia in patients with impaired renal function. Third, we used total ALP rather than bone ALP (BAP) as a bone turnover marker. Although BAP is more specific to bone formation than total ALP, in this study, we used total ALP to examine the effect of denosumab on bone turnover because of the small number of patients whose BAP was followed up after denosumab administration. However, hepatic ALP seemed not to significantly change in this study, because there were no notable changes in hepatic enzymes, such as aspartate aminotransferase, alanine aminotransferase, γ-glutamyl transpeptidase, and total bilirubin, 6 months after denosumab administration (data not shown). Most ALP is derived from the bone and liver^[26]^; therefore, the decrease in total ALP levels by denosumab in this study seem to reflect bone turnover suppression. Fourth, our study was retrospective, and the sample size was small. An a priori power analysis (α = 0.05, 2-tailed; desired power = 0.80) indicated that 16 paired observations were required to detect a mean within-subject calcium change of 0.5 mg/dL (SD of the difference 0.7 mg/dL). Our final sample comprised 15 patients, yielding an estimated power of 0.78, which may have limited our ability to detect smaller effects. Consequently, further large-scale prospective studies are warranted.

## 5. Conclusion

In this study, we demonstrated that osteoporosis treatment with denosumab alone in patients with PHPT, transiently decreased serum calcium levels, which returned to baseline at the next denosumab administration. Furthermore, no patient developed hypocalcemia, and BMD in the lumbar spine improved. These findings indicate that denosumab alone is a safe and effective treatment option for patients with PHPT who have not undergone parathyroidectomy.

## Author contributions

**Conceptualization:** Akira Okada, Ryuta Baba, Tsuguka Matsuda.

**Data curation:** Akira Okada.

**Formal analysis:** Akira Okada, Ryuta Baba.

**Investigation:** Akira Okada, Ryuta Baba.

**Methodology:** Ryuta Baba, Tsuguka Matsuda.

**Supervision:** Noboru Hattori, Haruya Ohno.

**Writing – original draft:** Akira Okada.

**Writing – review & editing:** Ryuta Baba, Yuria Ishibashi, Yu Otagaki, Takaya Kodama, Gentaro Egusa, Gaku Nagano, Tsuguka Matsuda, Noboru Hattori, Haruya Ohno.

## Supplementary Material
