# Supplementary material for: Effectiveness and safety of denosumab monotherapy in patients with primary hyperparathyroidism: A retrospective study
Source: Medicine (Baltimore). 2025 Oct 3;104(40):e44882. doi: 10.1097/MD.0000000000044882 (PMC12499689; doi:10.1097/MD.0000000000044882)
Supplement: Supplementary file 1 [file medi-104-e44882-s001.pdf]

Supplemental Table 1 - Baseline characteristics of patients whose serum calcium levels were followed up 3 months after denosumab administration

No	Age, years	Sex	The clinical department followed up after 3 months	Diseases
1	80	Female	Rheumatology and Clinical Immunology	Rheumatoid arthritis
2	73	Female	Rheumatology and Clinical Immunology	Rheumatoid arthritis and Sjögren's syndrome
3	83	Male	Hematology	Mantle cell lymphoma
4	77	Male	Digestive surgery	Postoperative of insulinoma
5	53	Female	Digestive surgery	Postoperative of NEN

NEN neuroendocrine neoplasm

Supplemental Table 2 - Comparison of baseline characteristics in terms of serum calcium levels measured 3 months after denosumab administration

	Measured 3 months after denosumab administration	Not measured 3 months after denosumab administration	*P* value
Serum calcium, mg/dL, median (IQR)	10.6 (10.3–10.9)	10.9 (10.6–11.0)	.38
ALP (JSCC), U/L, median (IQR)	321 (298–379)	287 (203–358)	.20
eGFR, mL/min/1.73 m^2, median (IQR)	68 (68–81)	68 (61–81)	.31

P-values were determined using an independent t-test. *Measured 3 months after denosumab* patients whose serum calcium levels were measured 3 months after denosumab administration, *Not measured 3 months after denosumab* patients whose serum calcium levels were not measured 3 months after denosumab administration, *IQR* interquartile range, *iPTH* intact parathyroid hormone, *ALP* alkaline phosphatase, *JSCC* Japanese Society of Clinical Chemistry, *eGFR* estimated glomerular filtration rate
